# 320. Heterogeneity in Clinical Presentations of Sepsis

**DOI:** 10.1093/ofid/ofad500.391

**Published:** 2023-11-27

**Authors:** Brett Biebelberg, Chanu Rhee, Caroline S McKenna, Michael Klompas

**Affiliations:** Massachusetts General Hospital, Philadelphia, Pennsylvania; Brigham and Women's Hospital, Boston, Massachusetts; Harvard Pilgrim Health Care Institute, Boston, Massachusetts; Harvard Medical School and Harvard Pilgrim Health Care Institute, Boston, Massachusetts

## Abstract

**Background:**

Sepsis treatment protocols stipulate a uniform care plan for all patients with possible sepsis. Sepsis clinical criteria, however, may capture a wide spectrum of illnesses. We sought to elucidate the heterogeneity of sepsis by characterizing the breadth of infection types, organ dysfunctions, and their differential outcomes in a large cohort of sepsis patients.

**Methods:**

We identified all adults admitted via the ED to 5 Massachusetts hospitals from 2015-2022 with possible sepsis, defined as suspected infection (blood culture orders and intravenous antibiotics) and organ dysfunction using CMS SEP-1 thresholds (drop in systolic blood pressure [SBP] ≥ 40mmHg or hypotension, elevated lactate, invasive or noninvasive ventilation, elevated creatinine or bilirubin, low platelets, or elevated INR/PTT). We identified infection sources using present-on-admission ICD-10 diagnosis codes and analyzed the frequency and mortality of each combination of infection site and organ dysfunction.

**Results:**

The cohort included 74,609 patients with suspected sepsis: median age 67 [IQR 55-79], 47% female, 24% non-white, 36% hypertension, 33% liver disease, and 28% chronic pulmonary disease. The overall in-hospital mortality rate was 9%. Patients varied widely in sources of infection (e.g. 33% pulmonary, 23% genitourinary, 13% intra-abdominal, and 1% obstetric/gynecologic) and organ dysfunctions (e.g. 57% drop in SBP ≥ 40mmHg, 9% hyperbilirubinemia, and 6% thrombocytopenia). Similarly, mortality rates varied from 3-13% across infection types (Figure 1) and from 3% (drop in SBP alone) to 27% (invasive ventilation) across organ dysfunctions (Figure 2). Combining infection types and organ dysfunctions further extended the spectrum of mortality from 0% for obstetric/gynecologic infections with thrombocytopenia to 35% for bacteremia with elevated INR or PTT (Figure 3).

**Figure 1:**
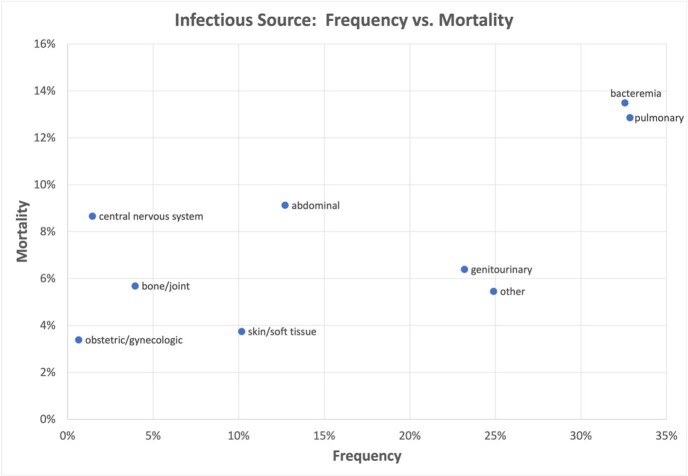
Scatter plot of frequency vs mortality of infectious sources in patients presenting with suspected sepsis

**Figure 2:**
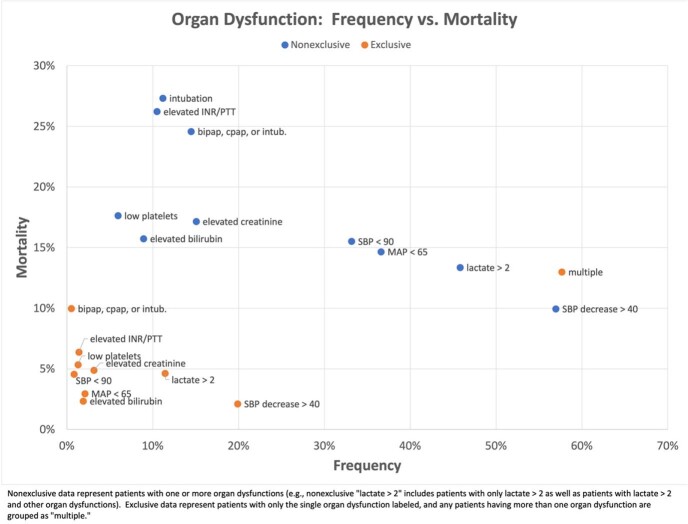
Scatter plot of frequency vs mortality of organ dysfunction patterns in patients presenting with suspected sepsis

**Figure 3:**
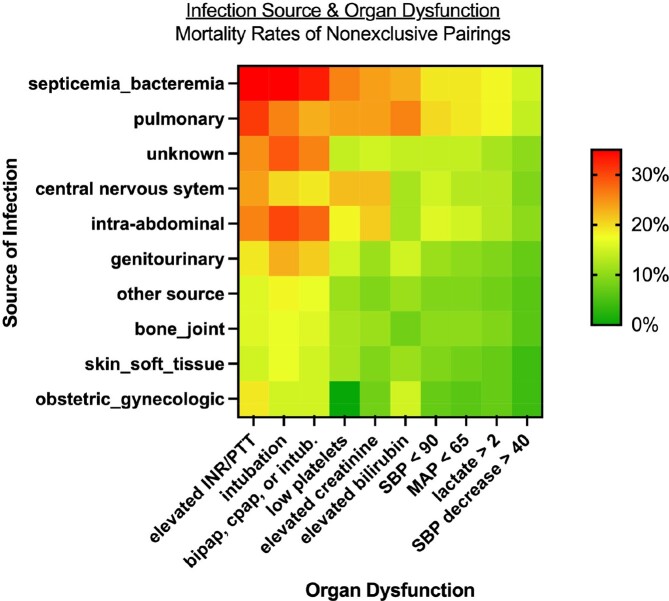
Heat map describing mortality rates for each combination of infectious source and organ dysfunction in patients with suspected sepsis

**Conclusion:**

Sepsis encompasses a wide spectrum of illnesses including multiple infection sites and organ dysfunctions that vary in crude mortality rates from 0-35%. The heterogeneity of sepsis begs the question whether “one-size-fits-all” bundles are suitable. Care tailored to each patient’s presentation may be more appropriate.

**Disclosures:**

**Chanu Rhee, MD, MPH**, Cytovale: Advisor/Consultant|Pfizer: Advisor/Consultant|UpToDate, Inc.: Honoraria **Michael Klompas, MD, MPH**, UpToDate, Inc.: Royalties for chapters on pneumonia

